# Donor age affects proteome composition of tenocyte-derived engineered tendon

**DOI:** 10.1186/s12896-018-0414-5

**Published:** 2018-01-16

**Authors:** Agnieszka J. Turlo, Yalda Ashraf Kharaz, Peter D. Clegg, James Anderson, Mandy J. Peffers

**Affiliations:** 1 Department of Pathology and Veterinary Diagnostics, Faculty of Veterinary Medicine, Warsaw University of Life Science, ul. Nowoursynowska 159c, 02-776 Warsaw, Poland; 2 Institute of Ageing and Chronic Disease, University of Liverpool, William Duncan Building, 6 West Derby Street, Liverpool, L7 8TX UK

**Keywords:** Engineered tendon, Tenocytes, Ageing, Label-free quantification

## Abstract

**Background:**

The concept of tissue engineering is to deliver to the injury site biological scaffolds carrying functional cells that will enhance healing response. The preferred cell source is autologous in order to reduce immune response in the treated individual. However, in elderly patients age-related changes in synthetic activity of the implanted cells and subsequent alterations in tissue protein content may affect therapeutic outcomes. In this study we investigated the effect of donor age on proteome composition of tenocyte-derived tendon tissue-engineered constructs.

**Results:**

Liquid chromatography tandem mass spectrometry was used to assess the proteome of tissue-engineered constructs derived from young and old equine tenocytes. Ageing was associated with altered extracellular matrix composition, especially accumulation of collagens (type I, III and XIV), and lower cytoskeletal turnover. Proteins involved in cell responsiveness to mechanical stimuli and cell-extracellular matrix interaction (calponin 1, palladin, caldesmon 1, cortactin) were affected.

**Conclusions:**

This study demonstrated significant changes in proteome of engineered tendon derived from young and old tenocytes, indicating the impact of donor age on composition of autologous constructs.

**Electronic supplementary material:**

The online version of this article (10.1186/s12896-018-0414-5) contains supplementary material, which is available to authorized users.

## Background

Ageing is associated with an increasing incidence of tendinopathies and impaired tendon repair following injury [1–3]. These events are thought to result from age-related modifications in tendon structure, especially extracellular matrix (ECM) content, accumulation of mechanically induced micro-trauma and decreased regenerative potential of tendon tissue [1, 4–7]. Tendon adaptability to loading conditions and its healing capacity depend predominantly on the cellular component, represented by tenocytes and tendon-derived stem cells (TDSCs) [8–11]. Tenocytes are tendon-specific fibroblasts, responsible for production and turnover of the ECM, while TDSCs are pluripotent cells capable of proliferation and differentiation into adult tenocytes [9, 12]. Current evidence suggests that natural tendon healing mechanism may be debilitated in chronic tendinopathies due to diminished proliferative potential of the native TDSCs [13–15]. TDSCs in tendinopathic tendon are also more likely to undergo aberrant differentiation into chondrocyte-like cells rather than tenocytes, further contributing to tendon degeneration by synthesis of inadequate ECM components [13, 14, 16, 17].

Tendon engineering combines use of mesenchymal stem cells (MSCs) or terminally differentiated fibroblasts and biocompatible scaffolds, providing functional grafts able to support reconstruction of the damaged tissue [18–21]. Similarly to cell-based therapies, autologous cell sources are preferred for tendon engineering to prevent host immune response against the implanted graft. Little is known on the impact of the donor’s age on the histological and biochemical properties of the tissue-engineered construct (TEC). Thorough knowledge of the age-related changes in TEC generated from different cell types is needed to provide rationale for choosing specific therapeutic option in older patients as well as selecting allogenic cell donors. Our recent study evaluating proteomic profiles of chondrogenic, osteogenic and tenogenic constructs derived from ageing human MSCs indicated differential expression of proteins involved in antioxidant protection and energy metabolism in old tendon TEC [22]. ECM matrisomal protein content was also affected, with higher numbers of neopeptides (indicating increased turnover) for the major ECM components identified in tenogenic constructs synthesized from old MSCs. No similar work has yet been undertaken for TEC generated from tenocytes. Tendon fibroblasts constitute a relevant source of cells used in tendon and ligament engineering [18, 23–25], therefore, investigating impact of donor age on composition of tenocyte-derived TEC appears to be of major importance. Several studies investigating the effects of ageing on the native tendon tissue reported alterations in ECM composition in older individuals. Increased degradation of type I collagen and decreased levels of selected proteoglycans and elastin were observed in older groups of equine superficial digital flexor tendon (SDFT) samples and rat Achilles tendon explants [4, 7, 26, 27]. It is not clear whether these changes are directly related to the decreasing ability of tendon cells to synthesize ECM proteins and/or enhanced release of matrix metalloproteinases (MMPs) degrading them [5, 6, 27, 28]. Findings from our previous study demonstrated that tissue engineered fibrin construct created from tenocytes derived from skeletally mature tendon shared similar characteristic with native tendon with regard to prominent ECM protein. However it is yet to be identified whether the ECM characteristics will be different in engineered tendon created from ageing tenocytes [29].

The horse is a recognised model for studying exercise- and age-related changes in tendon composition due to the functional similarity of equine and human energy-storing tendons and the relatively long lifespan of the species [30, 31]. Apart from studies analysing tendon ECM content, referenced above, equine SDFT has been used to investigate the effect of ageing on mechanical properties [3], response to fatigue loading [32] and collagen fibre conformation [33]. In the current study we continue work using equine tenocytes to investigate if the age of the tenocyte donor can affect the quality of tissue-engineered tendon contructs.

## Methods

The aim of this study was to perform global proteomic profiling of TEC, derived from equine tenocytes from young and old donors. We hypothesized that tenocyte ageing affects proteome of tendon TEC, and that characteristics of these changes would be similar to age-related processes observed in native tendon. Label-free relative quantification was employed to explore the impact of cell age on the abundance of intra- and extracellular proteins in engineered tendon.

### Sample collection

Equine tendons were harvested from the mid region of grossly normal superficial digital flexor tendons (SDFTs) from left forelimb of each horse, a single tendon per donor. Samples were collected as a by-product of the agricultural industry. Specifically, the Animal (Scientific Procedures) Act 1986, Schedule 2, does not define collection from these sources as scientific procedures. Ethical permission for tissue collection was obtained from the University of Liverpool Institute of Veterinary Sciences Research Ethics Committee (VREC352). Seven young donors (average age ± standard deviation; 4.4 ± 1.7 years) and six old donors (18 ± 2.4) were made into engineered tendon constucts as described below. The age of young horses was selected to ensure full skeletal maturity, whilst the old group included horses of greatest age obtainable with no abnormalities following gross tendon examination. Samples were acquired from breeds other than Thoroughbred racehorses due to high prevalence of pathology in tendons from such horses.

### Cell culture and tissue engineered construct formation

All chemicals are supplied by Sigma unless stated otherwise. All methods pertaining to tissue-engineered tendon have been previously described [22].

Equine tenocytes were isolated from the tendons on the day of collection and grown to passage 3 as described previously [15]. Briefly tendon without the paratendon or tendon sheath, was dissected and digested for 16 h at 37 °C in 1 mg/ml collagenase II. The cell suspension was strained and pelleted using centrifugation at 1932 G for 10 min. The cells were resuspended in complete Dulbecco’s modified Eagle’s medium (DMEM) supplemented with 10% foetal calf serum, penicillin (100 U/ml), streptomycin (100 mg/ml), and amphotericin B (2 mg/ml). Cells were seeded at 2.8 × 10^4^ cells/cm^2^ for construct set-up as previously described [34]. Briefly, each well of a six-well plate was coated with ~1.5 ml Sylgard (type 184 Silicone elastomer, WPI, Hertfordshire, UK) and pinned with silk sutures to create fixed anchor points. For creation of three technical fibrin constructs, 1.2 ml of 1.5 × 10^6^ cells/ml were suspended into 250 μl of 20 mg/ml fibrinogen and 25 μl of 200 U/ml thrombin (both Sigma-Aldrich, Dorset, UK) added. A volume of 480 μl of the mixture was immediately deposited in each well and vigorously shaken to ensure an even covering of the fibrin gel. Each cell- embedded fibrin gel was cultured in 2 ml phenol-red free DMEM supplemented with 100 U/ml penicillin/streptomycin, 10% FBS (Gibco, Paisley, UK), 500 ng/ml amphotericin, 2 mM L-glutamine (both life technologies, Paisley, UK) 200 μM l-ascorbic acid 2-phosphate (Sigma-Aldrich, Dorset, UK), non-essential amino acid (Sigma-Aldrich, Dorset, UK) at 10 μl/ml concentration, 0.02μg/ml human recombinant TGFβ-3 (Preprotech, London, UK) and aprotinin (Sigma-Aldrich, Dorset, UK) at 10 μl/ml. Engineered tendon constructs were incubated at 37 °C with 5% oxygen and harvested at 28 days.

### Protein extraction and sample preparation

Proteins were extracted from the constructs using 0.1% RapiGest™ [35]. Protein extracts were normalized following protein assay using the Bradford assay with Coomassie Plus™ protein assay reagent (Thermo Scientific, Rockford, IL) read at 660 nm. In-solution trypsin digestion was undertaken on all samples as described previously [7]. One sample from each group was excluded from further analysis due to poor protein extraction and the final number of biological replicates used in the study was 6 in young and 5 in old tenocyte group.

### Mass spectrometry and label-free quantification

Liquid chromatography tandem mass spectrometry (LCMS/ MS) was performed using a Ultimate 3000 Nano system (Dionex/Thermo Fisher Scientific) online The QExactive (Thermo-Scientific, Waltham, USA) as described previously [36].

For label-free quantification the Thermo raw files of the acquired spectra from in-solution tryptic digests were analysed by the ProgenesisQI™ software (Waters, Manchester, UK) [37]. Briefly, the top five feature spectra were exported from Progenesis-QI™ and utilized for peptide identification in PEAKS® 7 PTM (Bioinformatics Solutions Inc., Ontario, Canada) using Unihorse database. Search parameters used were: 10 ppm peptide mass tolerance and 0.01 Da fragment mass tolerance; one missed cleavage allowed; fixed modification, carbamidomethylation; and variable modifications, oxidation of methionine, and hydroxylation; 3 variable PTMs per peptide.

Proteins were identified with a false discovery rate (FDR) of 1% and a minimum of two unique peptides per protein. The resulting peptide-spectrum matches were imported into ProgenesisQI™ for label-free relative quantification. Differentially expressed (DE) proteins were defined with *p* < 0.05 and ±2-fold regulation.

The proteomics data were deposited to the ProteomeXchange Consortium [38] via the PRIDE partner repository with the dataset identifier PXD005322.

### Classification and functional network analysis of proteomics data

For structural and functional classification of identified proteins in both young and old TEC Ingenuity Pathway Analysis (IPA) [39] and Matrisome Project [40] were used. Networks functional analyses and gene ontology (GO) annotations of DE proteins were obtained from IPA and the Search Tool for Retrieval of Interacting Genes/Proteins (STRING) version 10.0 [41].

### Neopeptide identification

Neoeptides derived from extracellular matrix proteins were identified. The top five feature spectra previously exported from ProgenesisQI™ for protein identification were imported into an in-house Mascot server (Version 2.5.1) (Matrix Science, London, UK) and a ‘semi-tryptic’ peptide search carried out using the Unihorse database with results reimported into ProgenesisQI™. Search parameters used were: 10 ppm peptide mass tolerance and 0.01 Da fragment mass tolerance; one missed cleavage allowed; fixed modification, carbamidomethylation; and variable modifications, methionine, proline and lysine oxidation with FDR set to 1%.

The ProgenesisQI™ peptide measurements, together with the Unihorse FASTA file, were imported into a neopeptide Java application [42]. The application uses the FASTA protein database to identify the position of the peptide within the protein and together with assessment of the amino acids preceding and following the sequence, assigns peptides as either tryptic or as semi-tryptic/none-tryptic neopeptides.

The application normalizes neopeptide abundance through reducing the significance of protein variations across samples by summing the abundance of all tryptic peptides identified within the protein and subsequently calculating the ratio of semi-tryptic to tryptic peptide abundances for this protein. The application applied a t-test between young and old TEC groups with *p* < 0.05 considered significant. Neopeptide fold changes between the two groups were also calculated.

### Immunohistochemistry and biochemical analysis

Immunohistochemistry and biochemical analysis were used as validation techniques for the most DE proteins identified by LC-MS/MS. Tendon constructs were fixed in 4% paraformaldehyde for 48 h, longitudinally embedded in paraffin and cut into 4 μm thick sections on poly-L-lysine-coated slide. After endogenous peroxidase blocking (3% *w*/*v* H2O2), sections were pre-incubated in blocking solution (10% goat serum) for 1 h at room temperature and incubated overnight at 4 °C at 1/100 dilution with calponin antibody (ab46794, Abcam, UK). Sections were washed in phosphate buffered saline (PBS) three times before being incubated in Ztyochem Plus HRP Polymer anti-rabbit (ZUC032, Source Bioscience, UK) for 1 h at room temperature. After incubation, the sections were washed for three times for 5 min in TBS and incubated with appropriate of secondary antibody for 1 h at room temperature. Following washes in PBS immunostaining was detected with the chromogenic substrate DAB (SigmaFast 3,3-diaminobenzidine, Sigma, UK) and sections counterstained with Mayer’s Haemalum for 1 min and washed in tap water. Control experiments were carried out with omission of the primary antibody and substitution with non-immune rabbit IgG (Abcam). No staining was observed in the control experiments. Immunostained sections were visualized using Nikon eclipse 80i microscope. Intensity of stain was measured using Adobe Photoshop CC image processing program. Five biological replicates were analysed in each group.

Collagen content of tendon constructs (*n* = 7 in young and *n* = 6 in old group) were determined by hydroxyproline assay using previously described protocol [43].

### Statistical analysis

Statistical analysis performed for proteomic label-free dataset by ProgenesisQI™. Datasets for hydroxyproline and semi-quantitative analysis of immunohistochemistry results were tested for normality using Kolmogorov-Smirnov test (Graphpad Software, version 7, USA). All datasets were normally distributed and analysed using t-test. The significance level was at *p* < 0.05.

## Results

### Protein identification and ontology

**Fig. 1:**
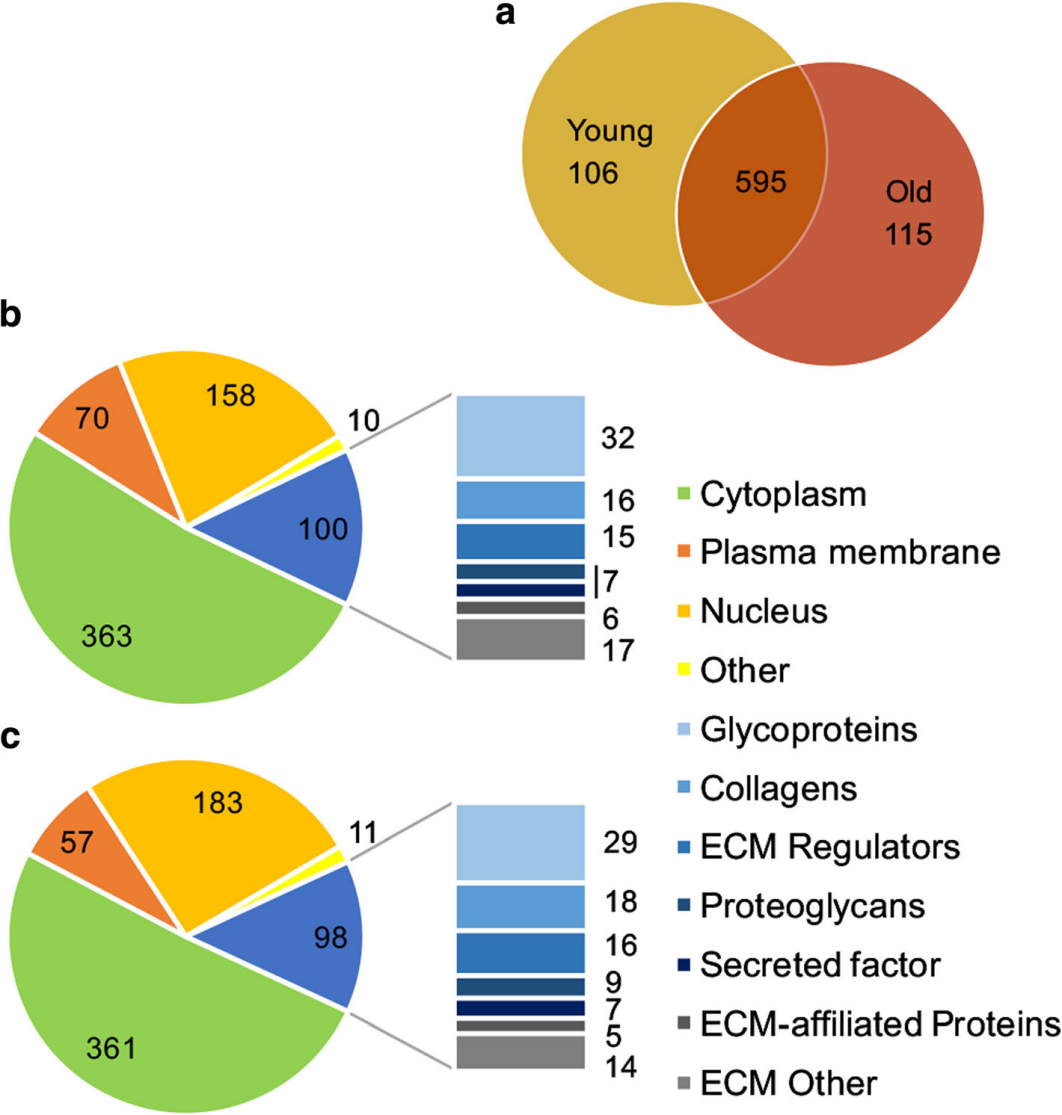
Number of proteins identified with PEAKS in engineered tendon-derived from young and old tenocytes. **a** Total number of proteins identified in respective age groups and common for both TEC types. Classification of all identified proteins was performed based on IPA and Matrisome Project and presented separately for young (*n* = 7) **(b)** and old (*n* = 6) **(c)** tenocyte TEC. The bar charts exhibit sublocations of proteins ascribed to extracellular matrix (ECM)

Total number of 6388 and 6492 peptides assigned to 701 and 710 proteins was identified in young and old tenocyte derived TEC by liquid chromatography tandem mass spectrometry (LCMS/MS). 595 proteins were common between young and old tenocyte TEC (Fig. 1a). Identified protein with ≥2 unique peptides, > 1% false discovery rate (FDR) and -10lgp >20 were considered as significant.

The dataset was transformed to a non-redundant gene identifier list of the respective human homologues, input into Ingenuity Pathway Analysis (IPA) and mapped for protein subcellular location. Proteins ascribed to the extracellular space were subjected to further categorization in Matrisome Project [40]. The undefined annotations where classified using UNIPROT. Categorized identified proteins in both young and old constructs proteins are presented in Fig. 1b, c and Additional file 1.

### Label-free relative quantification

To investigate the effect of cell ageing on TEC proteome composition, label-free quantification was undertaken on engineered tendon derived from young and old tenocytes using ProgenesisQI™. Proteins with a greater than 2-fold change in expression and *p* < 0.05 were considered differentially expressed (DE). Among 48 DE proteins 41 demonstrated higher abundance and 7 lower abundance in TEC from older donors. Proteins with the highest differential abundance in each age group are characterized in Table 1.

**Table 1:** Table of proteins showing the highest increase in abundance in young (*n* = 7) and old (*n* = 6) tenocyte-derived constructs, according to fold change

Highest mean condition	Accession (human)	Protein description	Function	Peptide count	Fold change	ANOVA (*p* value)
TEC derived from old cells	O94875	Sorbin and SH3 domain containing protein 2	Actin filament organization	10	5,1	0,02
	V9HWA5	Calponin 1	Actin binding, calmodulin binding	4	3,6	0,02
	P02461	Collagen type III alpha 1	ECM Collagen	38	3,5	0,01
	O43294	Transforming growth factor beta induced protein	ECM Glycoprotein	3	3,2	0,04
	Q05707	Collagen type XIV alpha 1	ECM Collagen	3	3,0	0,02
	P08123	Collagen type I alpha 2	ECM Collagen	48	3,0	0,00
	Q8WX93	Palladin, cytoskeletal associated protein	Enzyme	20	3,0	0,02
TEC derived from young cells	Q99623	Prohibitin 2	Transcription regulator	2	3,9	0,05
	P05106	Integrin beta 3	Transmembrane receptor	2	2,8	0,01
	P11166	Solute carrier family 2 member 1	Transporter	3	2,2	0,02
	P07919	Cytochrome b-c1 complex subunit 6	Enzyme	2	2,1	0,05
	P07093	Serpin family E member 2	ECM Regulator	3	2,1	0,04
	P08648	Integrin alpha 5	Transmembrane receptor	2	2,1	0,02
	P30049	ATP synthase subunit delta, mitochondrial	Enzyme	5	2,0	0,04

Only proteins with ≥2 unique peptides and *p* < 0.05 were presented. Abundant proteins in each group are also highlighted in Additional file 1

**Fig. 2:**
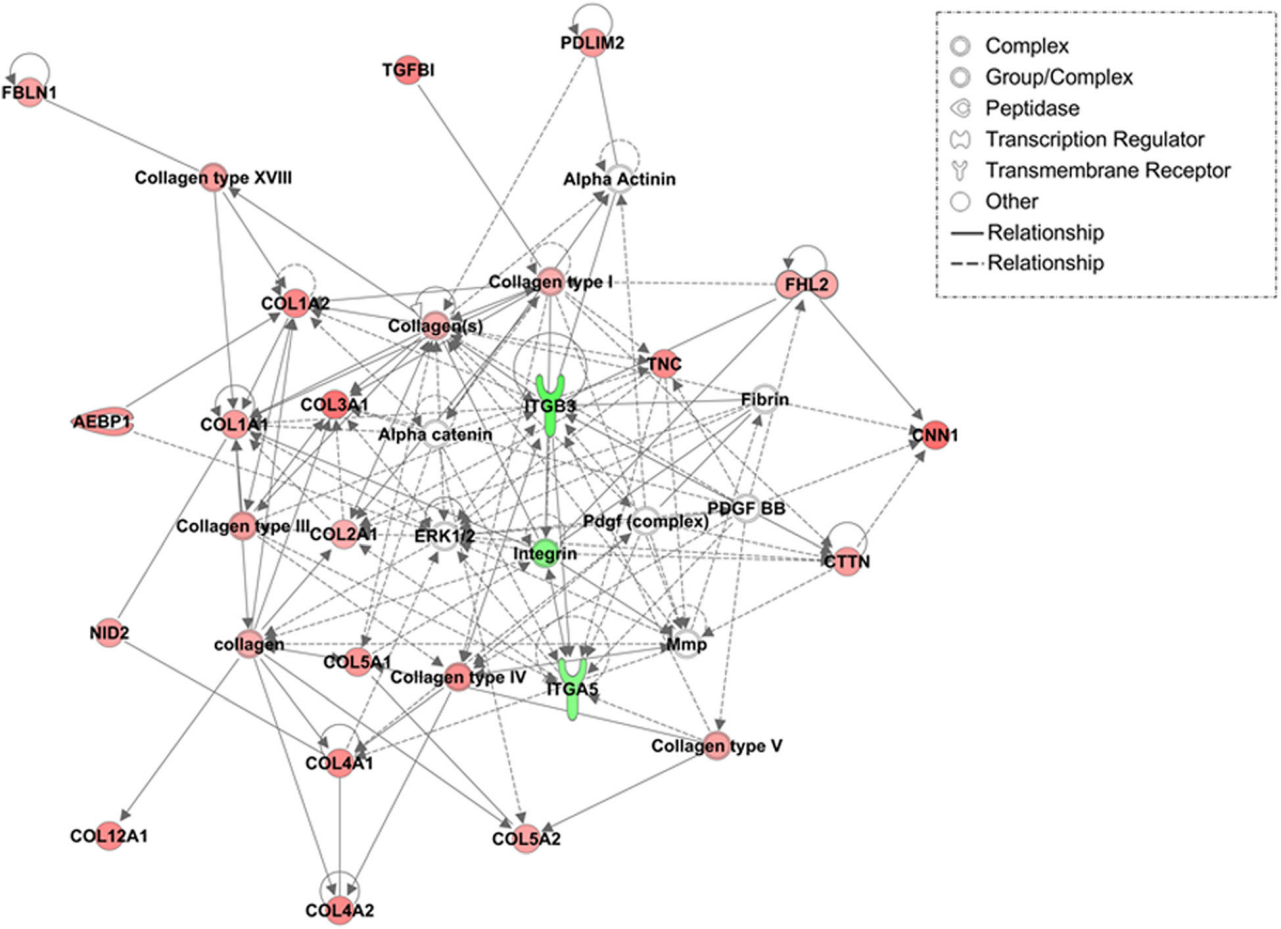
IPA top scoring network for proteins differentially abundant in TEC derived from young and old tenocytes. Proteins with higher abundance in old are displayed in red; with higher abundance in young – in green; with no difference in abundance between young and old – in white. Colour intensity is related to the increasing fold change in protein abundance between young and old constructs

**Fig. 3:**
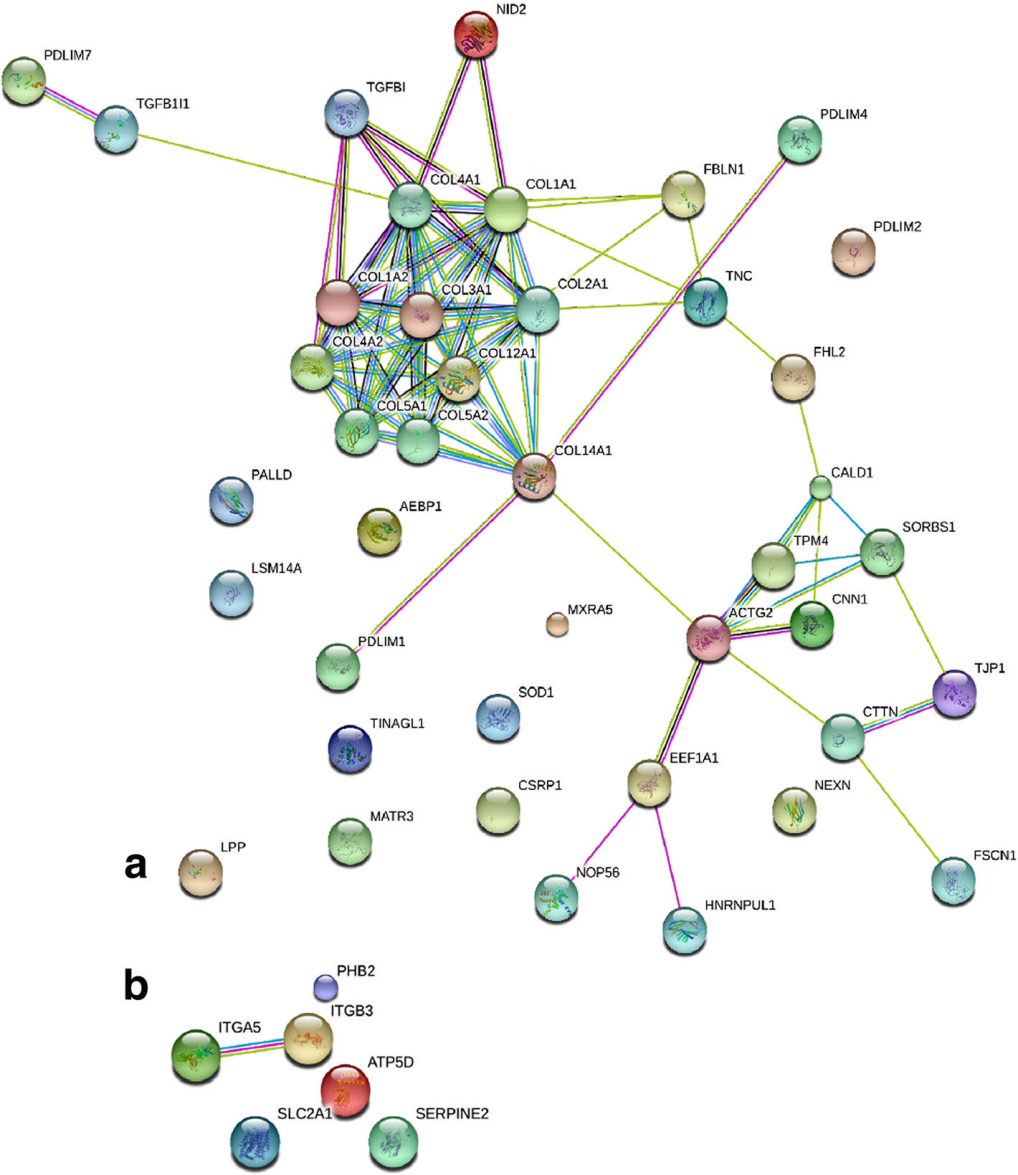
STRING interaction map of proteins differentially abundant between TEC from old (**a**) and young (**b**) tenocytes. **a** Two clusters of proteins are evident: ECM building collagens and intracellular structural protein involved in cytoskeleton formation. These two groups were also represented in proteins DE in older MSC constructs. Connections between the clusters signalize mechanical interaction of the cell and ECM, described by the GO annotation ‘focal adhesion’. The role of TGFBI in cell-collagen interaction is marked by multiple connections with different collagen subtypes. **b** Identified interactions involve integrins and respiratory chain enzyme subunits

Proteins DE between TEC derived from young and old tenocytes were scanned for functional networks and pathway analysis using IPA and the Search Tool for Retrieval of Interacting Genes/Proteins (STRING) [41]. One significant (IPA score > 40) network related to ‘connective tissue disorders’ was identified for proteins more abundant in old tenocyte TEC (Fig. 2). The most significant STRING GO annotations (FDR < 0.001) for proteins DE in old TEC were: ‘collagen catabolic processes’ and ‘extracellular matrix constituent’. STRING interaction map for proteins DE between old and young tenocyte TEC is shown in Fig. 3. Both IPA and STRING analyses of protein-protein interactions pointed to enhanced ECM turnover in old tenocyte TEC. Additionally, IPA identified transforming growth factor beta 1 (TGF-β1) as an upstream regulator of ECM metabolism (Additional file 2).

Top proteins enriched in the old TEC included cytoskeleton organization proteins (calponin 1, palladin), collagens (COL3A1, COL4A2, COL12A1) and transforming growth factor beta-induced protein (TGFBI) (Fig. 3, Table 1). STRING analysis determined the main GO cellular component for this group as ‘focal adhesion’ (FDR < 0.001).The proteins with higher abundance in young TEC, STRING revealed two subgroups based on protein-protein interactions: integrins (ITGB3, ITGA5) and mitochondrial respiratory chain enzymes (ATP synthase, cytchrome b-c1) (Fig. 3, Table 1).

### Immunohistochemistry and collagen content measurement

**Fig. 4:**
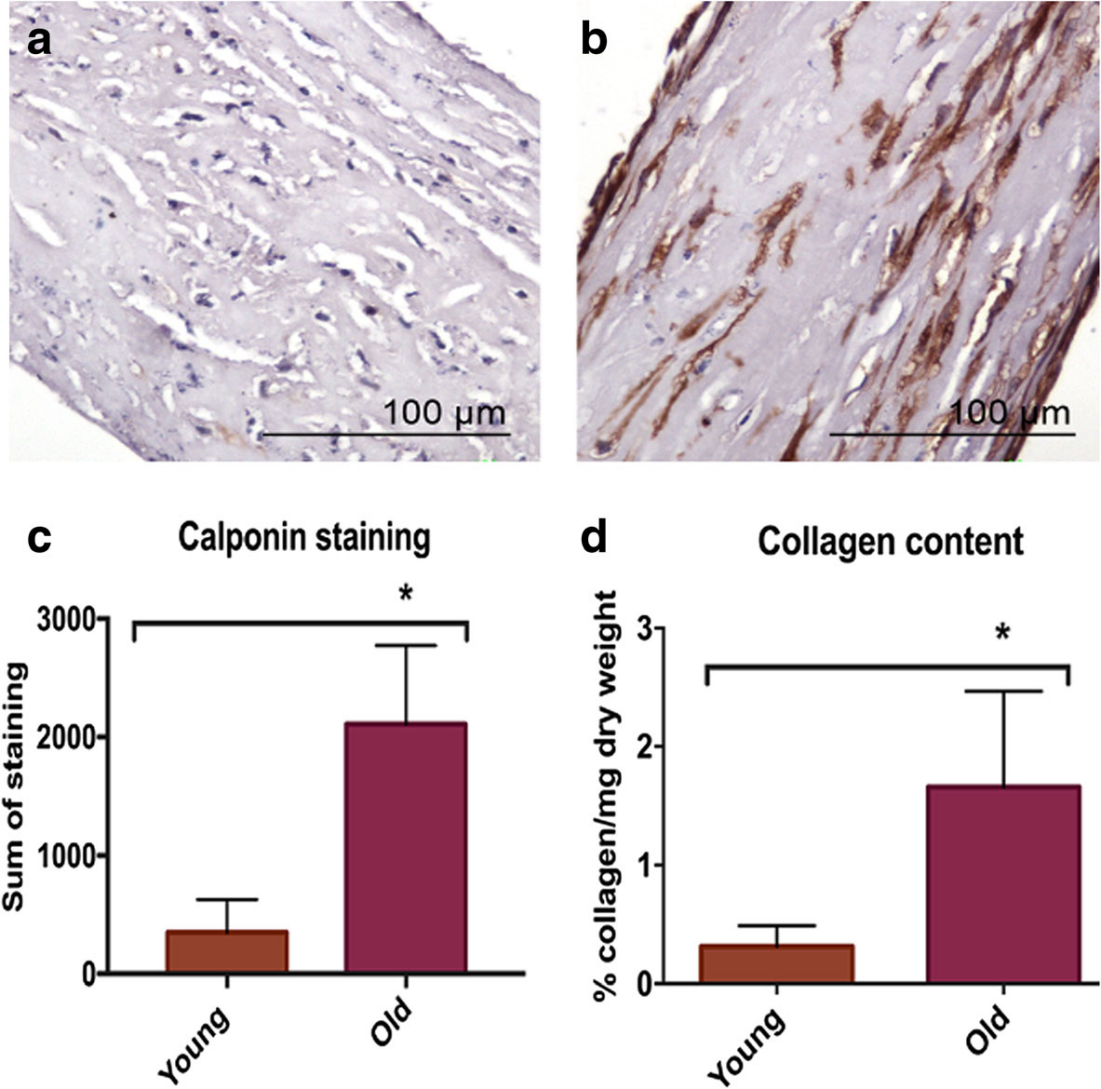
Immunohistochemistry and collagen content measurement. Immunostainning of calponin on young (*n* = 7) (**a**) and old (*n* = 6) (**b**) tendon constructs showed a marked staining in old construct. Significantly higher (* *p* < 0.05) intensity of calponin staining (**c**) and higher collagen content (**d**) was identified in old tendon constructs

TEC derived from older tenocytes demonstrated increased stain intensity of calponin protein (Fig. 4a and b). Semiquantitative analysis of calponin in young and old TEC resulted in significantly higher intensity of stain in older tenocyte derived TEC (*p* = 0.04) (Fig. 4c).

Collagen content was significantly (*p* ≤ 0.001) higher in older tenocyte-derived TEC comparing to young (mean ± standard deviation: 1,66 ± 0,8 and 0,31 ± 0,18, respectively) (Fig. 4d).

### Identification of ECM fragment patterns in engineered tendon

Neopeptides were identified in all samples (data not shown). There were significantly more neopeptides in engineered tendon derived from old tenocytes (*p* < 0.05). Neopeptides were identified for collagen XIIα1, collagen 1α1 and (Table 2).

**Table 2:** Differential collagen neoepitope abundances between tendon tissue-engineered constructs derived from young and old donors

Protein	Accession Number	Neopeptide Sequence	Previous Amino Acid	Following Amino Acid	Abundance Higher in Young or Old	Fold Change	*p* value
Collagen type I alpha 1 chain	F7D939	PQGPAGPAGPIGPVGAR	A	G	Old	0.7	0.008
Collagen type I alpha 2 chain	F6RTH9	GEAGAAGPAGPAGPRG	R	S	Old	0.6	0.045
		AGPVGAVGAPGPHGPVGPTGK	N	H	Old	2.0	0.001
		NGLQGLPGLAGQHGDQGAPGSVGPAGPR	H	G	Old	2.7	0.018
Collagen type XII alpha 1 chain	F6QD89	AIYPDESESDDLIGSERTPR	T	L	Old	5.4	0.001
		LEQLIPDTPYSVNIVAL	R	Y	Old	11.9	0.002
		LVQYSR	S	D	Young	3.1	0.021

## Discussion

This study describes the results of a comprehensive proteomic analysis of tendon TEC created utilising tenocytes from donors of different ages. The results support the hypothesis, that TEC proteome is dependent upon the age of the cells used for their creation. The most affected structural protein group was ECM, similarly to previous reports in ageing native tendon [7]. General limitation of this study was our lack of ability to estimate population doubling time once tenocytes were cultured in the construct setup. A different cell number within analysed constructs could affect protein content partitioning and subsequent results of proteomic analysis. However, the fact that only selected groups of proteins, intra- and extracellular, showed higher abundance in old tenocyte derived constructs supports the hypothesis that those changes could be attributed to ageing rather than size of cell population in the construct.

### ECM composition depends on age of tenocytes used for tissue engineering

Although the total number of proteins allocated to extracellular space did not differ significantly between the age groups, there was increase in number (Fig. 1b, c) and abundance (Table 1) of ECM collagens in older TEC. Increase in total collagen content was confirmed using a hydroxyproline assay. This novel finding is contradictory to other reports indicating that collagen synthesis remains unchanged in ageing native tendon and tenocyte culture [7, 26–28]. However, aberrations in collagen turnover leading to the accumulation of its partially degraded forms have been previously suggested to affect mechanical properties of ECM and contribute to the development of tendinopathies [26, 28]. In this study we observed significant increase in the number of neopeptides for collagenous proteins identified in old tenocyte TEC (Table 2). This was previously reported by our group in MSC derived engineered tendon [22] and suggests increased collagen turnover in TEC generated from older cells. Collagen metabolism depends on tendon fibroblasts, and their altered activity with age may lead to an accumulation of collagen breakdown products. The principle ontology terms for proteins DE in old tenocyte TEC were related to collagen catabolism and disorders of connective tissue. The most abundant collagen subunit identified in this age group was α2 chain of type III collagen (COL3A2). Increased proportions of type III collagen has been associated with tendinopathies and tendon injuries [44, 45] and tenocytes isolated from ruptured tendon were described to produce greater quantities of collagen III in an in vitro wound healing model [46]. Higher total collagen content in older TEC may be rather attributed to accumulation of degraded/abnormal collagen forms and impaired ECM-organising function of ageing tenocytes than increased fibrillogenesis.

One limitation of this study was that the engineered tendon was not subjected to mechanical testing; therefore the direct effect of cell ageing on the function of the created constructs could not have been evaluated. Based on literature, the age-related changes detected in TEC proteome are typically associated with declined tendon function and increased potential of injury [26, 44, 45, 47]. However, it would be interesting for the future studies to compare protein composition of the engineered tendon to its mechanical properties like stiffness, ultimate tensile strength and failure strain.

### TGFB1 regulates age-related changes in collagen expression

Transforming growth factor beta-1 (TGF-β1) was identified as potential upstream regulator of observed protein expression changes in old tenocyte derived TEC, most likely in relation to increased collagen-related proteins abundance. Although TGF-β is a well-established stimulator of collagen synthesis in response to mechanical loading in tendon [10, 48], its expression in tenocytes appears unaffected by age [6]. The cited study, however, utilized a rat model, which implicates significantly smaller age difference between examined groups comparing to human and equine studies [3, 4, 7, 22, 49]. In our previous studies TGF-β1 was proposed as the upstream regulator of age-related changes in cultured human MSCs [49] and MSC derived engineered tendon [22]. One of the top DE proteins in older TEC was transforming growth factor beta induced protein (TGFβI) considered to be regulatory for cell adhesion and cell-collagen interaction (Fig. 2). Given the strict relation between TGF-β1 activity and collagen synthesis, this finding appears to be in line with age-related changes in ECM collagen content described here, including the potential role of TGF-β1 in development of degenerative changes in tendon [48, 50].

### Cytoskeletal turnover is decreased in ageing tenocytes

The cytoskeleton has an important role in the mechanoresponsiveness of load-bearing tissues by mediating ECM strain to the cell nucleus and eliciting appropriate metabolic reaction [11]. Interaction between ECM and cytoskeleton occurs through focal adhesions and the intramembrane protein clusters that are also responsible for the maintenance of cell shape, and its ability to migrate. In this study, the most DE protein (increased in old cell based TEC) was the actin microtubule linking molecule, calponin 1 (Table 1). Calponins are a group of proteins that regulate interactions between F-actin, tropomyosin and calmodulin. They affect contractility of smooth muscle cells and participate in cytoskeleton organization in non-muscle cells [51–53]. Expression of calponins is regulated by mechanical tension exerted through extracellular environment and was demonstrated to increase in the cells cultured on stiff matrix [53, 54]. High abundance of calponin 1 in old tenocyte TEC may be related to changes in ECM content that affect mechanical properties of the engineered tissue and increase mechanical strain detected by the cells. Apart from calponin 1, other actin-binding molecules such caldesmon 1, cortactin and palladin were also identified. Transgelin, cytoskeletal protein previously referred to as cellular biomarker of ageing [55], demonstrated less than two-fold difference in abundance between young and old TEC. The main functional annotations for this group of proteins were ‘focal adhesion’ (STRING) and ‘cell movement’ (IPA, Additional file 2) which points to increased content of cytoskeletal components in older TEC. Increase in the content of intermediate filaments and cytoskeleton-associated proteins was observed in ageing tendon fibroblasts [7, 27, 51, 56, 57], MSC/TDSCs [13, 55] and MSC-based engineered tendon [22]. Accumulation of actin-related molecules was accompanied with disrupted cytoskeletal organization and redistribution of focal adhesions within the plasma membrane [13, 55–57]. These alterations in intracellular structure were considered to lead to the decrease in cell proliferation and their migration potential observed in vitro in tendon wound-healing models [27, 57] and MSC/TDSC cultures [13, 55, 58]. Similar changes in cytoskeletal components observed in our study may result in impaired ability of TEC generated from older cells to adapt to changing mechanical conditions and repair potential injuries. Proteins DE in young tenocyte-derived TEC also included molecules involved in the cell-ECM interaction, integrin beta-3 and alpha-5. These integrins are mainly receptors for glycoproteins, fibronectin and laminin, and not collagens that were significantly enriched in older TEC.

## Conclusions

This study evaluated for the first time proteomic profile of TEC synthesized from in vivo aged tenocytes. The results confirmed age-related changes previously observed in fibroblasts and MSCs; alterations in turnover of ECM collagens and cytoskeletal proteins. However, tenocyte TEC demonstrated ageing profile closer to native tissue than MSC derived engineered tendon, with the most characteristic change being the accumulation of collagenous protein and their breakdown products which may have detrimental consequence. Alterations in protein content of tenocyte TEC identified in this study may affect their function as tendon grafts. Our results contribute to the discussion on the efficiency of autologous cell therapies in elderly patients.

## Additional files

Additional file 1 All proteins identified by PEAKS in young and old tendon-derived TEC with correpsonding cellular sublocations defined by IPA and Matrisome Project. (XLSX 57 kb)

Additional file 2 Functional analysis and main pathways identified in proteins differentially expressed between engineered tendon derived from young and old tenocytes. (PDF 224 kb)

## Abbreviations

DE: Differentially expressed
ECM: Extracellular matrix
FDR: False discovery rate
IPA: Ingenuity pathway analysis
LC-MS/MS: Liquid chromatography tandem mass spectrometry
MMPs: Matrix metalloproteinases
MSCs: Mesenchymal stem cells
SDFT: Superficial digital flexor tendon
STRING: Search tool for retrieval of interacting genes/proteins
TDSC: Tendon-derived stem cells
TEC: Tissue-engineered constructs
TGF-β: Transforming growth factor beta
TGFβI: Transforming growth factor beta-induced protein
