# Supplementary material for: Donor age affects proteome composition of tenocyte-derived engineered tendon
Source: BMC Biotechnol. 2018 Jan 16;18:2. doi: 10.1186/s12896-018-0414-5 (PMC5771075; doi:10.1186/s12896-018-0414-5)
Supplement: Supplementary file 2 — Functional analysis and main pathways identified in proteins differentially expressed between engineered tendon derived from young and old tenocytes. (PDF 224 kb) [file 12896_2018_414_MOESM2_ESM.pdf]

INGENUITY
PATHWAY ANALYSIS

Analysis Name: teno old to young ipa - 2016-07-15 04:32 PM
Analysis Creation Date: 2016-07-15
Build version: 389077M
Content version: 27821452 (Release Date: 2016-06-14)

Analysis Settings

Reference set: Ingenuity Knowledge Base (Genes Only)
Relationship to include: Direct and Indirect
Includes Endogenous Chemicals
Optional Analyses: My Pathways My List

Filter Summary:
Consider only relationships where
confidence = Experimentally Observed

(c) 2000-2016 QIAGEN. All rights reserved.

Top Canonical Pathways

Name	p-value	Overlap
Hepatic Fibrosis / Hepatic Stellate Cell Activation	2.95E-10	4.9 % 9/183
Intrinsic Prothrombin Activation Pathway	5.44E-07	13.8 % 4/29
Agrin Interactions at Neuromuscular Junction	1.85E-05	5.8 % 4/69
Dendritic Cell Maturation	7.14E-05	2.6 % 5/190
Atherosclerosis Signaling	2.01E-04	3.1 % 4/127

Top Upstream Regulators

Upstream Regulator	p-value of overlap	Predicted Activation
HRAS	1.07E-18	Inhibited
TGFB3	1.34E-16	
TGFB1	1.09E-15	Activated
TP53	7.02E-15	Activated
AHR	7.77E-14	Inhibited

Top Diseases and Bio Functions

Diseases and Disorders

Name	p-value	#Molecules
Cancer	6.74E-03 - 8.54E-19	45
Connective Tissue Disorders	6.83E-03 - 8.54E-19	21
Organismal Injury and Abnormalities	6.83E-03 - 8.54E-19	45
Reproductive System Disease	6.12E-03 - 8.54E-19	36
Endocrine System Disorders	6.23E-03 - 5.41E-18	25

Molecular and Cellular Functions

(c) 2000-2016 QIAGEN. All rights reserved.

INGENUITY PATHWAY ANALYSIS

Name	p-value	#Molecules
Cellular Assembly and Organization	6.83E-03 - 4.34E-12	27
Cellular Function and Maintenance	4.75E-03 - 4.34E-12	27
Cellular Movement	6.83E-03 - 7.33E-09	27
Cell-To-Cell Signaling and Interaction	6.83E-03 - 1.84E-07	18
Cell Death and Survival	6.83E-03 - 4.30E-07	21

Physiological System Development and Function

Name	p-value	#Molecules
Cardiovascular System Development and Function	6.83E-03 - 8.03E-08	19
Organismal Development	6.83E-03 - 6.16E-07	20
Embryonic Development	6.83E-03 - 5.10E-06	17
Tissue Morphology	6.83E-03 - 5.10E-06	19
Connective Tissue Development and Function	6.83E-03 - 6.95E-06	20

Top Tox Functions

Assays: Clinical Chemistry and Hematology

Name	p-value	#Molecules
Decreased Levels of Albumin	1.14E-02 - 1.14E-02	1
Increased Levels of ALT	1.36E-02 - 1.36E-02	1
Increased Levels of Hematocrit	1.99E-02 - 1.99E-02	2
Increased Levels of Red Blood Cells	2.23E-02 - 2.23E-02	2

Cardiotoxicity

Name	p-value	#Molecules
Congenital Heart Anomaly	1.06E-01 - 2.28E-03	2
Cardiac Infarction	3.59E-02 - 6.83E-03	3

(c) 2000-2016 QIAGEN. All rights reserved.

INGENUITY PATHWAY ANALYSIS

Cardiac Arteriopathy	4.25E-02 - 1.36E-02	3
Cardiac Dysfunction	6.63E-02 - 1.44E-02	2
Cardiac Arrythmia	2.47E-01 - 4.91E-02	2

Hepatotoxicity

Name	p-value	#Molecules
Liver Hyperplasia/Hyperproliferation	1.16E-01 - 5.49E-04	26
Liver Cirrhosis	2.03E-02 - 2.04E-03	4
Hepatocellular Carcinoma	1.16E-01 - 2.87E-03	7
Liver Fibrosis	5.83E-03 - 4.18E-03	4
Liver Proliferation	5.83E-03 - 5.83E-03	2

Nephrotoxicity

Name	p-value	#Molecules
Renal Inflammation	2.29E-01 - 2.28E-03	2
Renal Nephritis	2.29E-01 - 2.28E-03	2
Renal Damage	1.91E-02 - 1.91E-02	2
Renal Tubule Injury	1.91E-02 - 1.91E-02	2
Nephrosis	3.81E-02 - 3.81E-02	1

Top Regulator Effect Networks

ID	Regulators	Diseases & Functions	Consistency Score
1	LEP	organismal death,size of body	3.13
2	TNF	leukocyte migration	1.225
3	AHR	organismal death	-12.017

(c) 2000-2016 QIAGEN. All rights reserved.

INGENUITY PATHWAY ANALYSIS

Top Networks

ID	Associated Network Functions	Score
1	Cancer, Connective Tissue Disorders, Organismal Injury and Abnormalities	48
2	Cell Morphology, Connective Tissue Development and Function, Cellular Movement	39
3	Cell Death and Survival, Cell Morphology, Hematological System Development and Function	18
4	Cell-To-Cell Signaling and Interaction, Skeletal and Muscular System Development and Function, Organ Morphology	11

Top Tox Lists

Name	p-value	Overlap	
Hepatic Fibrosis	3.00E-09	6.9 %	7/101
Genes associated with Chronic Allograft Nephropathy (Human)	1.44E-05	14.3 %	3/21
Recovery from Ischemic Acute Renal Failure (Rat)	4.56E-04	14.3 %	2/14
Persistent Renal Ischemia-Reperfusion Injury (Mouse)	2.13E-03	6.7 %	2/30
Acute Renal Failure Panel (Rat)	8.85E-03	3.2 %	2/62

Top Analysis-Ready Molecules

Exp Fold Change up-regulated

Molecules	Exp. Value	Exp. Chart
**SORBS2**	↑ 5.144	
**CNN1**	↑ 3.579	
**COL3A1**	↑ 3.461	
**TGFBI**	↑ 3.160	
**COL14A1**	↑ 3.040	
**COL1A2**	↑ 2.997	
**PALLD**	↑ 2.953	
**COL4A2**	↑ 2.940	

(c) 2000-2016 QIAGEN. All rights reserved.

**TNC**	↑ 2.922	
**NEXN**	↑ 2.901	

Exp Fold Change down-regulated

Molecules	Exp. Value	Exp. Chart
**PHB2**	↓ -3.923	
**ITGB3**	↓ -2.803	
**SLC2A1**	↓ -2.224	
**UQCRH**	↓ -2.142	
**SERPINE2**	↓ -2.108	
**ITGA5**	↓ -2.096	
**ATP5D**	↓ -2.005	

(c) 2000-2016 QIAGEN. All rights reserved.

INGENUITY PATHWAY ANALYSIS
